# The future of gene-edited crops in China

**DOI:** 10.1093/nsr/nwac063

**Published:** 2022-04-01

**Authors:** Jian-Kang Zhu

**Affiliations:** Institute of Advanced Biotechnology and School of Life Sciences, Southern University of Science and Technology, China; Editorial Board Member of NSR

Hunger has been a major issue throughout human history and sadly remains so today. Although China has the largest population, its arable land per capita ranks no better than 126th among the >190 countries in the world. Thanks to the widespread adoption of modern agricultural technologies including the use of improved crop varieties, chemical fertilizers and pesticides, the Chinese people have enjoyed an increasing abundance of food. Nevertheless, grain security remains a major challenge for China and the country must import nearly 100 million tons of soybean and tens of millions of tons of corn and other cereal grains each year. To further improve living standards while reducing grain import, China has been extraordinarily supportive of agricultural research in general and of breeding in particular, and is determined to see technological breakthroughs in the seed sector.

As a result of that strong support, agricultural research has flourished in China. For example, scientists in China have published more papers in recent years concerning crop genomics and plant gene-editing technologies than their peers in any other country. The CRISPR/Cas gene-editing technology is revolutionizing the field of crop breeding because of its precision, speed and simplicity in generating the desired genetic mutations needed for crop improvement. The research of scientists in China and elsewhere has shown that gene editing can help breed crops that yield higher, that are more nutritious and more tolerant of extreme weather, and that require fewer chemical fertilizers and pesticides [1]. Now, the gene-editing tool can be used not only to remove or weaken genes unfavorable for agronomic traits but also to boost the numerous genes that are beneficial for crops [2].

To use gene-edited crops in agriculture, regulatory approvals are required. The gene-editing tool in essence is a biological mutagen that generates genetic mutations in plants, much like the chemical mutagens and radiations used in conventional mutagenesis breeding. After the gene-editing tool is removed from the edited plants through genetic segregation, transgene-free gene-edited crops can be obtained. Transgene-free gene-edited crops are not different from conventional crops in that they all harbor mutations that can occur naturally, and they therefore do not present any added food-safety or environmental risks compared to crops bred by traditional mutagenesis breeding. Consistently with this characteristic, gene-edited crops cannot be distinguished from conventional crops, whereas transgenic crops (GMOs) can be easily discerned based on the presence of transgenes. In many countries such as the USA and Japan, transgene-free gene-edited crops are treated as conventional crops and are exempt from the restrictive policies applied to GMOs by government regulatory agencies. China's Ministry of Agriculture and Rural Affairs has recently issued a guideline for the regulatory approval of gene-edited crops, thus paving the way for commercialization of this critically important crop-breeding technology. According to this guideline, transgene-free gene-edited crops are still managed under the policy umbrella of GMOs, but may require much less complicated food and environmental safety evaluations compared to true GMOs.

To feed the human population in the face of climate change, natural disasters and pandemics, China and the whole world need gene-edited crops, more so now than ever before. Researchers as well as farmers are all looking forward to seeing gene-edited crops approved quickly and grown in China and elsewhere to contribute to sustainable agriculture and grain security.
